# Identification of a Selective Inhibitor of Human NFS1, a Cysteine Desulfurase Involved in Fe-S Cluster Assembly, via Structure-Based Virtual Screening

**DOI:** 10.3390/ijms26062782

**Published:** 2025-03-19

**Authors:** Zhilong Zhu, Haisheng Gan, Yanxiong Wang, Guanya Jia, Heng Li, Zhiwei Ma, Jun Wang, Xiaoya Shang, Weining Niu

**Affiliations:** School of Life Sciences, Northwestern Polytechnical University, Xi’an 710072, China; zhilongzhu_2019@mail.nwpu.edu.cn (Z.Z.); hsgan@mail.nwpu.edu.cn (H.G.); wangyanxiong2023@mail.nwpu.edu.cn (Y.W.); jiagy@mail.nwpu.edu.cn (G.J.); aheng@mail.nwpu.edu.cn (H.L.); zwma@mail.nwpu.edu.cn (Z.M.); wangj@nwpu.edu.cn (J.W.); shangxy@nwpu.edu.cn (X.S.)

**Keywords:** cysteine desulfurase (NFS1), virtual screening, inhibitor, ferroptosis, Fe-S cluster, molecular docking

## Abstract

Human cysteine desulfurase (NFS1) participates in numerous critical cellular processes, including iron–sulfur (Fe-S) cluster biosynthesis and tRNA thiolation. NFS1 overexpression has been observed in a variety of cancers, and thus it has been considered a promising anti-tumor therapeutic target. To date, however, no inhibitors targeting NFS1 have been identified. Here, we report the identification of the first potent small-molecule inhibitor (Compound **53**, PubChem CID 136847320) of NFS1 through a combination of virtual screening and biological validation. Compound **53** exhibited good selectivity against two other pyridoxal phosphate (PLP)-dependent enzymes. Treatment with Compound **53** inhibited the proliferation of lung cancer (A549) cells (IC_50_ = 16.3 ± 1.92 μM) and caused an increase in cellular iron levels due to the disruption of Fe-S cluster biogenesis. Furthermore, Compound **53**, in combination with 2-AAPA, an inhibitor of glutathione reductase (GR) that elevates cellular reactive oxygen species (ROS) levels, further suppressed the proliferation of A549 cells by triggering ferroptotic cell death. Additionally, the key residues involved in the binding of the inhibitor to the active center of NFS1 were identified through a combination of molecular docking and site-directed mutagenesis. Taken together, we describe the identification of the first selective small-molecule inhibitor of human NFS1.

## 1. Introduction

Cysteine desulfurase, an essential pyridoxal phosphate (PLP)-dependent enzyme [1], catalyzes the desulfurization of L-cysteine to generate alanine and a protein-bound cysteine persulfide (-SSH) intermediate on the active site cysteine residue [2]. The persulfide sulfur can be further transferred directly to other sulfur-containing biomolecules, such as iron–sulfur (Fe-S) clusters, thiamine, lipoic acid, the molybdenum cofactor (Moco), and thiolated nucleosides in tRNA [3,4,5]. In humans, cysteine desulfurase (NFS1) is the ortholog of *Escherichia coli* IscS and *Azotobacter vinelandii* NifS and plays a crucial role in mitochondrial Fe-S cluster biosynthesis. The biosynthetic pathways of Fe-S clusters are highly conserved from prokaryotes to mammals from an evolutionary perspective. Fe-S clusters are ancient protein prosthetic groups that participate in a variety of biological processes, including redox catalysis, electron transfer, DNA repair, substrate binding and activation, and tRNA modification. Defects in Fe-S cluster synthesis have now been associated with many human diseases, such as Friedreich’s ataxia and ISCU myopathy [6].

In humans, Fe-S cluster-containing proteins are located in the mitochondria, cytosol, and nucleus. To date, it is believed that most Fe-S clusters are synthesized in the mitochondria. The core machinery required for the de novo biosynthesis of Fe-S clusters in the mitochondria is a multiprotein supercomplex containing the cysteine desulfurase NFS1, frataxin (FXN), the scaffold protein ISCU, the accessory protein ISD11, and the acyl carrier protein ACP [7]. NFS1 catalyzes the removal of sulfur from the substrate L-cysteine and transfers the sulfur (as a persulfide group) to a cysteine residue, Cys381, located in a mobile loop of NFS1. This loop connects the active site of NFS1 with the Fe-S cluster assembly site on the ISCU surface [8]. ISCU is the major scaffold protein upon which the transient Fe-S clusters are initially assembled and from which they are then delivered to recipient proteins [9]. FXN interacts with ISCU and NFS1 to facilitate the activity of NFS1 as an allosteric activator, participating in sulfur transfer chemistry [10,11,12]. ISD11 is a small protein exclusively found in eukaryotes, with no obvious equivalent in prokaryotes, and plays a key role in assisting and stabilizing NFS1. The function of ACP is not well understood, but it can interact with ISD11 [13,14]. The functional dimeric structure of NFS1 is stabilized by two ISD11-ACP heterodimeric units.

The role of Fe-S cluster biogenesis in carcinogenesis remains unclear. However, the expression levels of several genes involved in Fe-S biogenesis are altered in tumor tissues compared to their normal tissue counterparts, according to the TNMplot database [15]. Indeed, the overexpression of NFS1 has been associated with the progression of various cancers, including lung cancer, colon cancer, gastric cancer, and pancreatic cancer. Alvarez et al. reported that NFS1 lies in a region of genomic amplification present in lung adenocarcinoma, and the suppression of NFS1 cooperates with the inhibition of cysteine transport to trigger ferroptosis in vitro and slow tumor growth [16]. Lin et al. revealed that the loss of NFS1 significantly enhanced the sensitivity of colorectal cancer cells to oxaliplatin, and that NFS1 deficiency, when combined with oxaliplatin, triggered PANoptosis by increasing intracellular levels of reactive oxygen species (ROS) [17]. Chafe et al. confirmed that the depletion of NFS1 or the blocking of cyst(e)ine availability by inhibiting the cystine–glutamate antiporter xCT enhanced ferroptosis and significantly inhibited tumor growth [18]. Recently, Mao et al. found that NFS1 was upregulated in gastric cancer tissues, which can be effectively diagnosed and dynamically monitored to evaluate the prognosis of gastric cancer patients [19]. These studies strongly suggest that NFS1 may be an attractive target for cancer therapeutics, and the development of small-molecule inhibitors of NFS1 could be a promising anticancer treatment strategy.

To date, unfortunately, very few cysteine desulfurase inhibitors have been reported. Previous studies have indicated that D-cycloserine, a second-line drug against *Mycobacterium tuberculosis* in clinical use, could inhibit the cysteine desulfurase activity of *Plasmodium falciparum* with an IC_50_ value of ~29 μM [20]. Analysis of the inhibition mechanism showed that D-cycloserine binds to the PLP cofactor, forming an adduct with enzyme-bound PLP and thereby inhibiting the enzyme [20,21]. Recently, eprenetapopt (APR-246), an anticancer agent targeting mutant p53, was shown to inhibit Fe-S cluster biogenesis by limiting the cysteine desulfurase activity of human NFS1, likely through diminishing the availability of free cysteine by directly conjugating with cysteine [22]. However, as millimolar concentrations were required to inhibit NFS1, APR-246 was clearly not a potent inhibitor of human NFS1. Currently, the inhibition of NFS1 is primarily achieved through RNA interference (RNAi)-induced gene silencing, and no potent inhibitors targeting human NFS1 have been identified. Therefore, it is urgent to develop novel, potent inhibitors for studying the biological function of human NFS1 and for treating cancers.

In the present study, to obtain potent and selective inhibitors of human NFS1, a method for the combination of structure-based virtual screening and biological evaluation was employed to screen a small-molecule compound library. Subsequent similarity-based analog searching resulted in the identification of Compound 53 (PubChem CID 136847320), the first selective small-molecule inhibitor targeting human NFS1. Further cellular experiments revealed that treatment with Compound 53 significantly inhibited the proliferation of lung cancer (A549) cells and caused an increase in cellular iron levels due to the disruption of Fe-S cluster biogenesis. Therefore, Compound 53 could be used to explore the biological roles of NFS1 and serve as a lead compound for the design of therapeutic agents for the treatment of lung cancer.

## 2. Results

### 2.1. Structure-Based Virtual Screening

**Figure 1:**
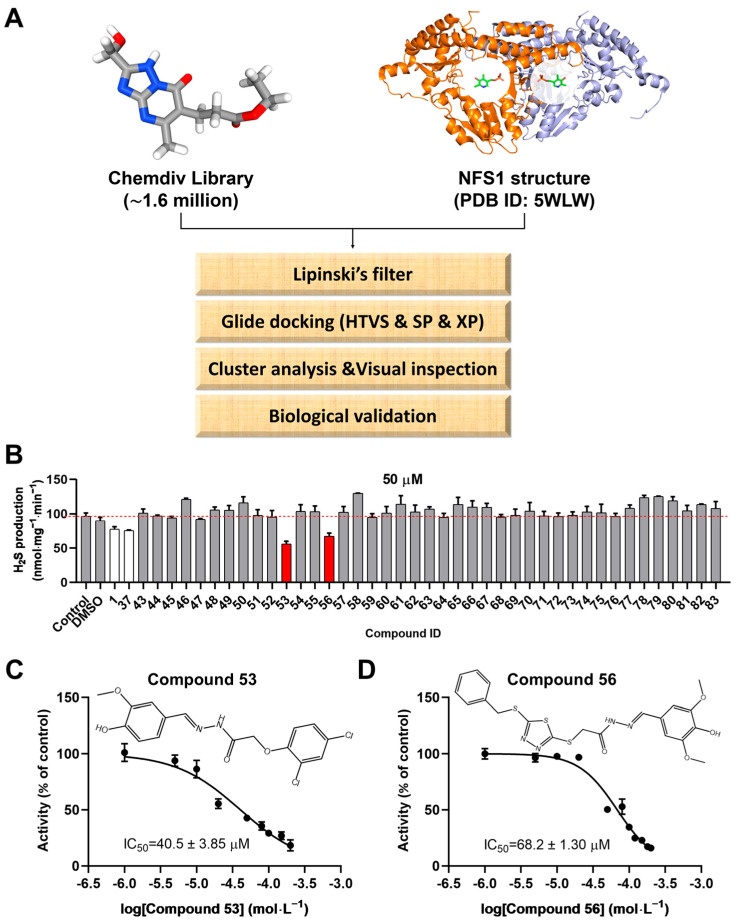
Virtual screening procedures and activity assays for NFS1 in vitro. (**A**) Schematic representation of the virtual screening procedure (the cofactor PLP in the NFS1 crystal structure is highlighted within a white circle). (**B**) Evaluation of the effects of compounds 43–83, which were generated based on a pharmacophore model constructed from the skeletons of Compound **1** (PubChem CID 135504454) and Compound **37** (PubChem CID 99733644), on NFS1 activity at a final concentration of 50 μM (n = 4). Compound **1** and Compound **37**, which were screened from the initial 42 candidate compounds (Figure S2B), were used as controls. (**C**,**D**) Effects of Compound **53** (PubChem CID 136847320) and Compound **56** (PubChem CID 136911182) at various concentrations on NFS1 activity. The chemical structures of Compounds **53** and **56** are shown in the inset of Figure 1C,D, respectively. Data are presented as the means ± SD (n = 4).

A crystal structure of human NFS1 in complex with the cofactor PLP (PDB ID: 5WLW) was selected for structure-based virtual screening. To identify candidate compounds targeting human NFS1, we employed a computational docking method to simulate NFS1–compound binding and screened compounds from the commercial ChemDiv library. As shown in Figure 1A, the prepared ligands were docked into the defined docking grids of NFS1 using the Glide module. Ultimately, 283 compounds were retained (Figure S1A) and clustered into 20 groups (Figure S1B) for visual inspection. From each cluster, at least one molecule was selected based on visual inspection to ensure broader chemical space coverage, thereby improving the hit rate. As a result, 42 compounds with diverse scaffolds were purchased from TargetMol (Shanghai, China) for biochemical assays.

### 2.2. Assessment of Human NFS1 Inhibitory Activity of Compounds from Virtual Screening

The 42 candidate compounds were tested for their inhibitory effects on human NFS1 using an in vitro H_2_S-producing assay. First, human NFS1 was expressed and purified in *Escherichia coli*, and the recombinant human NFS1Δ1-55 was used for subsequent experiments (Figure S2A). Subsequently, the effects of the selected compounds (100 µM) on human NFS1 activity were evaluated. The results revealed that two compounds (Compound **1** and Compound **37**) exhibited an approximately 50% inhibition of NFS1 at 100 µM (red bars in Figure S2B), with IC_50_ values of 153.2 ± 3.42 µM and 140.6 ± 3.49 µM, respectively (Figure S2C,D). The chemical structures of Compound **1** (PubChem CID 135504454) and Compound **37** (PubChem CID 99733644) are shown in the inset of Figure S2C,D, respectively.

Next, to validate the scaffolds of Compounds **1** and **37**, as well as to identify analogs with higher inhibitory activity, a similarity-based analog search was conducted in the ChemDiv library using a pharmacophore model constructed from the skeletons of Compounds **1** and **37**. A total of 41 analogs of Compounds **1** and **37** were selected for biochemical evaluation using the H_2_S-producing inhibition assay described above. Among them, two compounds (Compound **53** and Compound **56**) exhibited an approximately 40%–50% inhibition of NFS1 activity at 50 μM (red bars in Figure 1B), with IC_50_ values of 40.5 ± 3.85 µM and 68.2 ± 1.30 µM, respectively. Furthermore, Compounds **53** and **56** were found to inhibit NFS1 activity in a dose-dependent manner (Figure 1C,D). The chemical structures of Compounds **53** and **56** are shown in the inset of Figure 1C,D, respectively. To further elucidate the inhibition type of Compounds **53** and **56**, enzyme kinetic analyses were performed using varying concentrations of the inhibitors and the substrate L-cysteine (Figure S3). The results revealed that as the concentration of the compounds increased, the *K*_m_ value increased while the *V*_max_ remained almost unchanged, indicating that Compounds **53** and **56** act as competitive inhibitors of NFS1. The inhibition constants (*K*_i_) were calculated to be 6.4 ± 0.48 µM and 9.6 ± 0.57 µM, respectively (Table S1).

To evaluate the selectivity of Compounds **53** and **56**, their effects on the activities of two other PLP-dependent enzymes, human cystathionine-β-synthase (CBS) and cystathionine-γ-lyase (CSE), were determined using a H_2_S-producing assay. Human CBS and CSE were expressed and purified in *E. coli* with a purity exceeding 90%, as confirmed by SDS-PAGE (Figures S4A and S5A). Subsequently, the H_2_S-producing activities of CBS and CSE were assessed in the presence of different concentrations of Compounds **53** and **56**. A widely used CBS inhibitor, AOAA, and a specific CSE inhibitor, PAG, were used as positive controls (Figures S4B and S5B). The results showed that Compounds **53** and **56** had no significant inhibitory effects on CBS and CSE at a concentration of 100 µM (Figures S4C,D and S5C,D), suggesting that Compounds **53** and **56** exhibited good selectivity towards NFS1.

### 2.3. Compound 53 and Compound 56 Suppressed the Proliferation of Lung Cancer Cells

**Figure 2:**
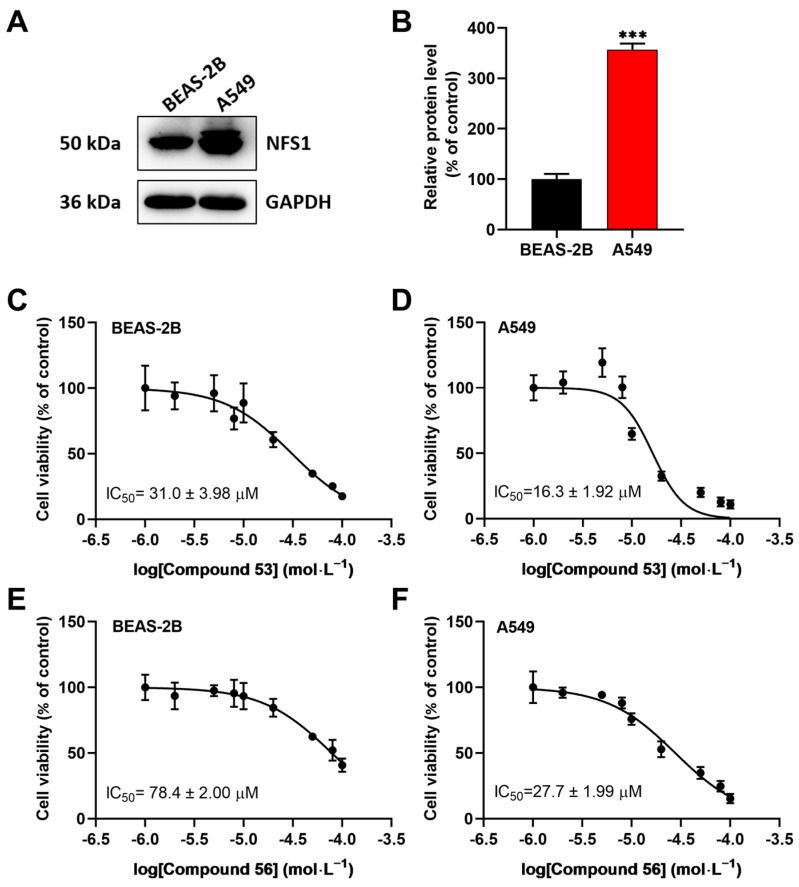
The effects of Compounds **53** and **56** on the proliferation of normal human bronchial epithelial cells (BEAS-2B) and lung adenocarcinoma cells (A549). (**A**,**B**) The expression levels of NFS1 in BEAS-2B and A549 cells, respectively (n = 3). (**C**,**D**) The cell viability assays of BEAS-2B and A549 cells treated with varying concentrations of Compound **53** for 72 h (n = 6). (**E**,**F**) The cell viability assays of BEAS-2B and A549 cells treated with varying concentrations of Compound **56** for 72 h (n = 6). Data are presented as the means ± SD. *** *p* < 0.001.

Since recent studies have demonstrated that NFS1 is upregulated in lung cancer cells [16,23,24], we speculated that these two compounds might suppress the proliferation of A549 cells by inhibiting NFS1 activity. First, the expression levels of NFS1 in BEAS-2B normal lung epithelial cells and A549 lung cancer cells were determined. The results showed that NFS1 expression was significantly elevated in A549 cells compared to BEAS-2B cells (Figure 2A,B). Next, we evaluated the anti-proliferative activity of Compounds **53** and **56** in A549 and BEAS-2B cells using the CCK-8 assay. As shown in Figure 2C–F, Compound **53** suppressed the viability of BEAS-2B and A549 cells with IC_50_ values of 31.0 ± 3.98 µM and 16.3 ± 1.92 µM, respectively (Figure 2C,D), while Compound **56** suppressed the viability of BEAS-2B and A549 cells with IC_50_ values of 78.4 ± 2.00 µM and 27.7 ± 1.99 µM, respectively (Figure 2E,F). In addition, both Compound **53** and Compound **56** inhibited cell proliferation in a dose-dependent manner and displayed significantly higher inhibitory activity against A549 cancer cells than against BEAS-2B normal cells. Since Compound **53** exhibited stronger inhibitory activity against A549 cells compared to Compound **56**, it was selected for further investigation.

### 2.4. Compound 53 Inhibited the Activity of the Fe-S Proteins ACO and SDH in A549 Cells

**Figure 3:**
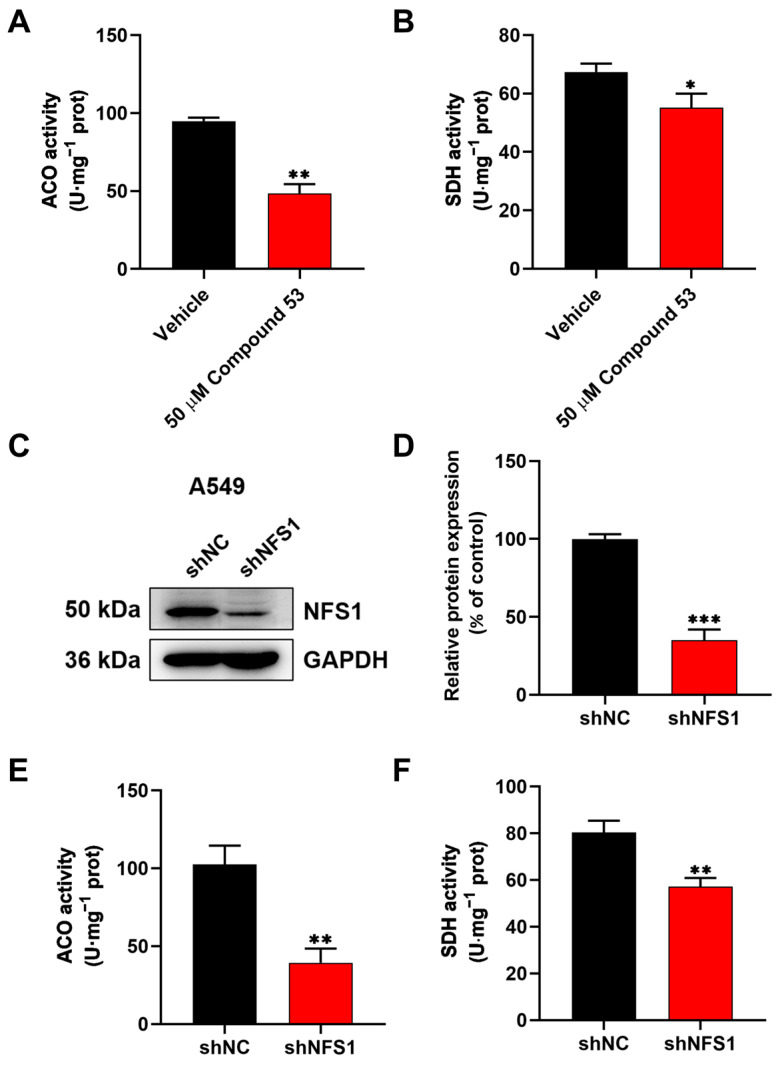
Compound 53 inhibits the activities of aconitase (ACO) and succinate dehydrogenase (SDH) in A549 cells. (**A**,**B**) The effect of Compound 53 (50 μM) on the activities of aconitase (ACO) and succinate dehydrogenase (SDH) in A549 cells, with the vehicle (DMSO) as a negative control (n = 3). (**C**,**D**) NFS1 was knocked down in A549 cells using small interfering RNA (siRNA), and GAPDH was used as a loading control. The quantification of relative NFS1 expression in NFS1-knockdown (shNFS1) A549 cells (n = 3). The expression levels of NFS1 in A549 cells (shNC) were set to 100%. (**E**,**F**) The determination of the activities of aconitase (ACO) and succinate dehydrogenase (SDH) in NFS1-knockdown (shNFS1) A549 cells (n = 3). Data are presented as the means ± SD. * *p* < 0.05. ** *p* < 0.01. *** *p* < 0.001.

Previous findings suggest that NFS1 suppression leads to an insufficient supply of Fe-S clusters, which subsequently affects the activities of intracellular Fe-S proteins [25,26]. To investigate the effects of Compound **53** on Fe-S cluster biosynthesis via NFS1 inhibition, we measured the activities of two Fe-S cluster-containing enzymes, aconitase (ACO) and succinate dehydrogenase (SDH), in A549 cells. The results revealed that treating A549 cells with 50 µM of Compound **53** decreased the activities of ACO and SDH to 51.3% and 81.8%, respectively, compared to the control group (Figure 3A,B). To assess whether Compound **53** specifically targeted NFS1 in cells, NFS1 was knocked down in A549 cells using small interfering RNA (siRNA). As shown in Figure 3C,D, the protein expression level of NFS1 decreased to approximately 30% of that in the control group. Consequently, the activities of ACO and SDH were significantly reduced in NFS1-knockdown cells (Figure 3E,F), consistent with the observations in Compound **53**-treated cells. These results indicate that Compound **53** likely inhibits the activities of ACO and SDH in cells by disrupting Fe-S cluster biosynthesis through the direct inhibition of NFS1 activity.

### 2.5. Compound 53 Treatment Increased Cellular Iron Levels

**Figure 4:**
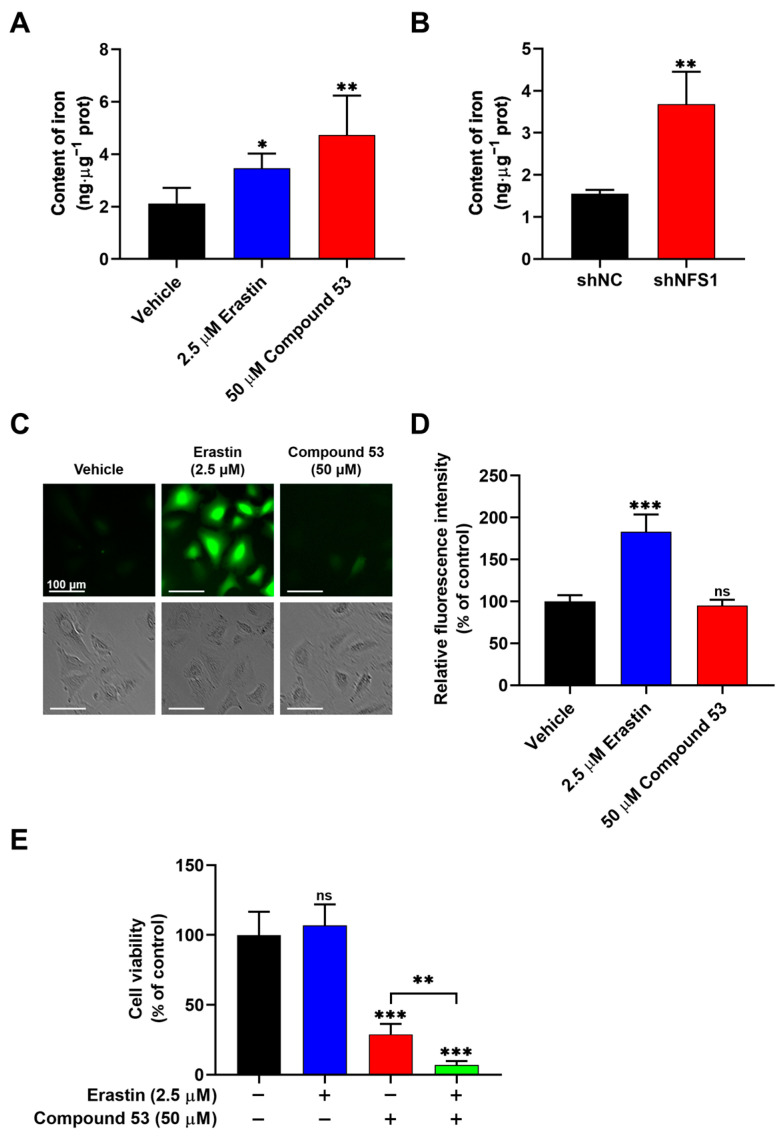
Compound **53** elevates the iron content but does not affect the ROS levels in A549 cells. (**A**) Determination of cellular iron content in A549 cells treated with Erastin (2.5 μM) or Compound **53** (50 μM). The vehicle (DMSO) was used as a negative control (n = 3). (**B**) Iron content in A549 cells (shNC) and NFS1-knockdown (shNFS1) A549 cells (n = 3). (**C**) Fluorescent images of A549 cells stained with DCFH-DA (green) (×20 magnification). The cells were incubated with Erastin (2.5 μM) or Compound **53** (50 μM) and then imaged. Scale bar = 100 μm. (**D**) Quantification of fluorescence intensities for ROS signaling, with data from panel C for comparison. The graph represents the relative fluorescence intensity compared with the untreated cells (n = 6). (**E**) Cell viability of A549 cells treated with Erastin (2.5 μM, blue bar), Compound **53** (50 μM, red bar), or both (green bar). The cell viability of the untreated cells was set to 100%. Data are presented as the means ± SD (n = 6). * *p* < 0.05. ** *p* < 0.01. *** *p* < 0.001, ns: no significant.

Previous studies have reported that the suppression of NFS1 can activate the iron starvation response, leading to increased iron absorption by cells [27,28,29]. To assess the effect of Compound 53 on cellular iron levels, we measured the total iron content in A549 cells treated with 50 µM of Compound **53** and 2.5 µM of Erastin (a ferroptosis inducer), respectively. As shown in Figure 4A, treatment with Compound **53** significantly increased the cellular iron content, similar to the effect of the well-recognized ferroptosis inducer, Erastin. Increased cellular iron levels were also observed in NFS1-knockdown A549 cells (Figure 4B), consistent with previous reports [26]. These results suggested that treatment with Compound **53** produced a similar effect to NFS1 gene silencing in A549 cells. However, unlike cells exposed to Erastin, treatment with Compound **53** did not significantly affect intracellular ROS levels (Figure 4C,D).

Next, we sought to determine the effects of combining Compound 53 and Erastin on cell viability. Since 2.5 µM of Erastin did not significantly affect cell viability (Figure S6A), we examined the effect of Compound **53** (50 µM) on the proliferation of A549 cells, either in the presence or absence of 2.5 µM of Erastin. The results indicated that the anti-proliferative activity of Compound **53** increased remarkably in the presence of 2.5 µM of Erastin (Figure 4E). Considering that treatment with 2.5 µM of Erastin significantly increased intracellular ROS levels (Figure 4C,D), we speculated that the elevated cellular iron and ROS levels, induced by Compound **53** and Erastin (2.5 µM), respectively, might trigger ferroptotic cell death.

### 2.6. Compound 53 Synergized with 2-AAPA to Induce Ferroptosis

**Figure 5:**
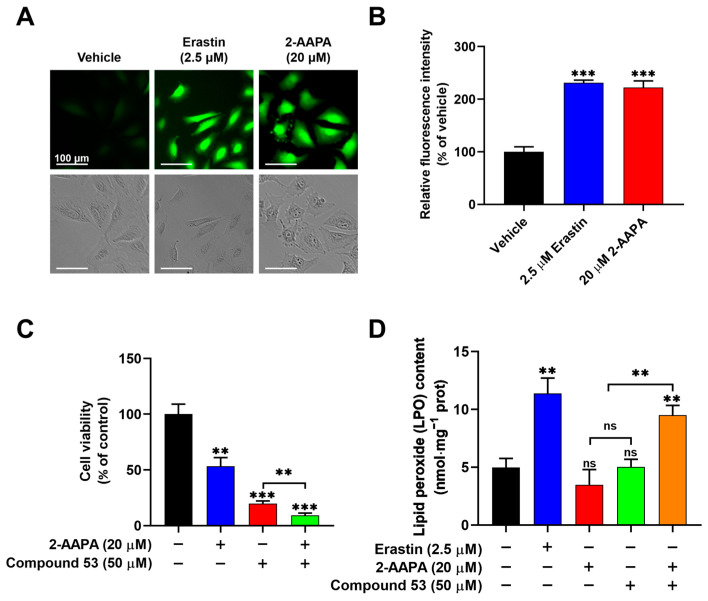
Compound **53**, in combination with the glutathione reductase (GR) inhibitor 2-AAPA, triggers ferroptosis. (**A**) Fluorescent images of A549 cells stained with DCFH-DA (green) (×20 magnification). The cells were incubated with Erastin (2.5 μM) or 2-AAPA (20 μM) and then imaged. Scale bar = 100 μm. (**B**) Quantification of the fluorescence intensities of ROS signaling, with data from panel A for comparison. The graph represents the relative fluorescence intensity compared with untreated cells (n = 6). (**C**) The cell viability of A549 cells treated with 2-AAPA (20 μM, blue bar), Compound **53** (50 μM, red bar), or both (green bar). The cell viability of untreated cells (black bar) was set to 100% (n = 6). (**D**) Determination of the lipid peroxide (LPO) levels in A549 cells incubated with Erastin (2.5 μM, blue bar), 2-AAPA (20 μM, red bar), Compound **53** (50 μM, green bar), and the combination of 2-AAPA (20 μM) and Compound 53 (50 μM) (orange bar), respectively (n = 4). The untreated cells (black bar) were set as the control. Data are presented as the means ± SD. ** *p* < 0.01. *** *p* < 0.001, ns: no significant.

We subsequently investigated whether treatment with Compound **53** in combination with an ROS inducer, 2-AAPA (an irreversible inhibitor of glutathione reductase (GR)) [30], could induce ferroptosis in A549 cells. First, intracellular ROS levels in A549 cells treated with 2-AAPA (20 µM) and Erastin (2.5 µM) were measured using a fluorescent probe. The results indicated that both 2-AAPA and Erastin significantly elevated cellular ROS levels (Figure 5A,B). Additionally, 2-AAPA inhibited the proliferation of A549 cells with an IC_50_ value of approximately 20 μM (Figure S6B). Compared to the anti-proliferative activity of Compound **53** (50 μM) or 2-AAPA (20 μM) alone, the combination of Compound **53** and 2-AAPA greatly enhanced the inhibitory effect on cell proliferation (Figure 5C).

To evaluate whether the combination of Compound **53** and 2-AAPA induced ferroptotic cell death, we measured intracellular lipid peroxide (LPO) levels, a well-recognized indicator of ferroptosis. As shown in Figure 5D, compared to the elevated LPO levels observed in cells treated with Erastin (2.5 µM), no significant changes in intracellular LPO levels were observed following treatment with either Compound **53** (50 µM) or 2-AAPA (20 µM) alone. However, the combination of Compound **53** and 2-AAPA remarkably increased LPO levels in A549 cells. Collectively, these results demonstrated that elevated levels of cellular iron and ROS, induced by Compound **53** and 2-AAPA, respectively, can cause cell death via ferroptosis.

### 2.7. Key Residues Involved in the Compound 53 Binding Site on NFS1

**Figure 6:**
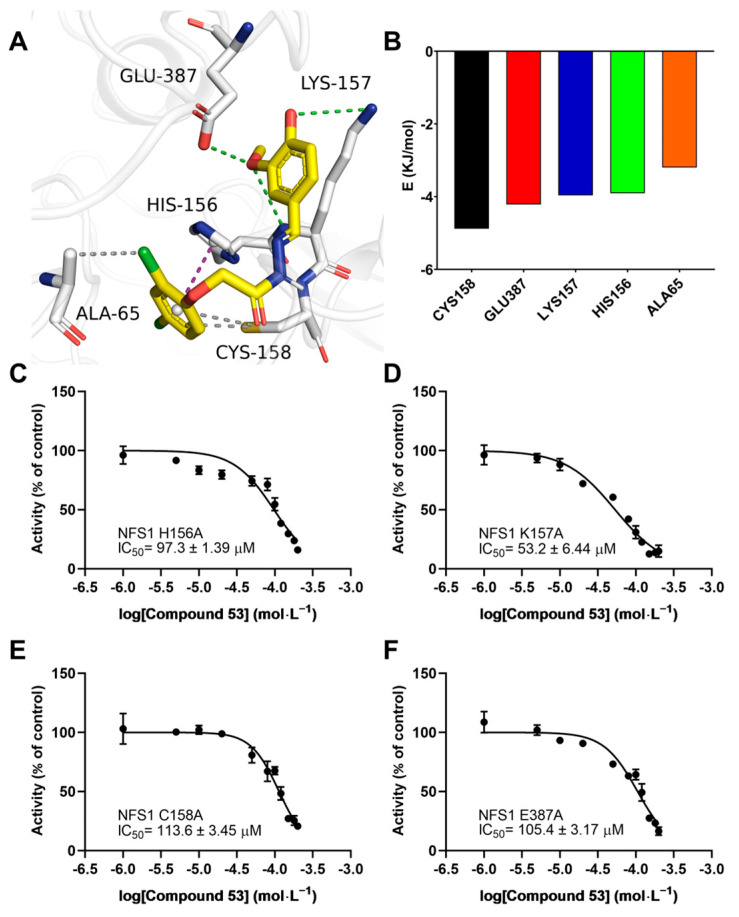
Interaction analysis of the simulated NFS1–Compound **53** complex. (**A**) A snapshot from the simulated Compound **53**–NFS1 complex at the end of a 200 ns molecular dynamics (MD) simulation. Key amino acid residues involved in the interaction between Compound **53** and NFS1 are shown. Green represents hydrogen bonds, magenta denotes π-π stacking interactions, and gray indicates hydrophobic interactions. (**B**) Total energy contribution of each amino acid residue in the NFS1 protein. (**C**–**F**) Dose–response curves of Compound **53** with the H156A NFS1, K157A NFS1, C158A NFS1, and E387A NFS1 mutants (n = 4). Data are presented as the means ± SD.

To elucidate the binding mode of Compound **53** with NFS1, we analyzed the docking poses generated by AutoDock Vina, followed by a 200 ns molecular dynamics (MD) simulation. As shown in Figure S7A, during the MD simulation, the RMSD values of the NFS1 backbone ranged from 0.3 to 0.4 nm, while those of Compound **53** ranged from 0.2 to 0.35 nm, indicating that the system had reached equilibrium, and that Compound **53** could bind stably to NFS1. As shown in Figure 6A, Compound **53** appears to bind at the active center of NFS1 through multiple binding interactions. The total binding free energy (Δ*G*_bind_) calculated using the MM-PBSA method was −83.48 ± 0.903 kJ/mol for the Compound **53**–NFS1 complex, with van der Waals forces being the major contributing factor, as indicated by a Δ*G*_bind_ value of −206.48 ± 0.812 kJ/mol (Figure S7B). According to the binding free energy calculation, five amino acid residues (Cys158, Glu387, Lys157, His156, and Ala65) contributed more than 3 kJ/mol to the total interaction energy. These five residues, listed in descending order of their total binding energy contributions, were predicted to be involved in Compound **53** binding to NFS1 in the simulated Compound **53**–NFS1 complex (Figure 6A,B). In this structure, the hydrogen bonds (Glu387 and Lys157), hydrophobic interactions (Cys158 and Ala65), and π-π stacking interactions (His156) play crucial roles in the structural stability of the Compound **53**–NFS1 complex.

To further validate the accuracy of the simulated results, the residues Cys158, Glu387, Lys157, and His156 were mutated to alanine using site-directed mutagenesis. The specific activities of the wild-type and the four mutant NFS1 proteins are shown in Figure S7C. The H_2_S-producing activity of the K157A and E387A mutants was similar to that of wild-type NFS1, while the activity of the H156A mutant decreased dramatically. The activity of the C158A mutant was approximately two-fold greater than that of wild-type NFS1, consistent with a previous report [1]. Next, we evaluated the effect of Compound **53** on the activities of these four NFS1 mutants by determining their IC_50_ values. Compared with the high affinity of Compound **53** for wild-type NFS1, which had an IC_50_ value of 40.5 ± 3.85 μM, these four NFS1 mutants caused a significant decrease in the binding affinity of Compound **53** to varying degrees, with IC_50_ values ranging from 53.2 ± 6.44 μM to 113.6 ± 3.45 μM (Figure 6C–F). These results indicate that these residues likely play a critical role in the interaction between Compound **53** and NFS1, consistent with the total binding energy contributions determined in the molecular docking analysis.

## 3. Discussion

Recent studies have revealed the involvement of human NFS1 in the pathogenesis of various malignancies, highlighting its potential as a target for cancer therapy [24,31]. The development of potent and highly selective inhibitors targeting NFS1 is considered a promising therapeutic strategy for cancer treatment [17]. However, to date, there have been few reports of compounds that act as NFS1 inhibitors. In this study, Compound **53** was identified as a novel and selective inhibitor of human NFS1 through in silico screening. To the best of our knowledge, Compound **53** is the first reported small-molecule inhibitor of human NFS1.

Based on the enzyme activity assays, Compound **53** significantly inhibited NFS1 activity in a dose-dependent manner. Further selectivity assays demonstrated that Compound **53** exhibited high selectivity against two other PLP-dependent enzymes, CBS and CSE, which are responsible for the biogenesis of H_2_S in cells. In addition, Compound **53** showed significantly higher inhibitory activity against A549 cancer cells compared to BEAS-2B normal cells (Figure 2C,D). One possible mechanism for this is that the higher expression level and enzyme activity of NFS1 are required in A549 cancer cells, as evidenced by Western blot analysis (Figure 2A,B). Thus, the inhibition of NFS1 activity by Compound **53** resulted in stronger inhibitory effects on A549 cancer cells than on BEAS-2B normal cells. However, it should be noted that Compound **53** may have additional targets in cells beyond the NFS1 protein.

The activities of two Fe-S cluster-containing proteins, aconitase (ACO) and succinate dehydrogenase (SDH), were inhibited in A549 cells treated either with Compound **53** or transfected with siRNA-targeting NFS1 (Figure 3). This finding is consistent with previous reports that defects in Fe-S cluster biosynthesis impair the enzymatic activities of ACO and SDH [26]. In addition to affecting the biological functions of Fe-S cluster-containing proteins, defects in Fe-S cluster biosynthesis also disrupt cellular iron homeostasis, leading to an iron-starvation phenotype [9]. Indeed, a previous study indicated that the inhibition of NFS1 using siRNA disrupted Fe-S cluster biosynthesis, activated iron-starvation responses, and triggered ferroptosis [16]. As expected, treatment with Compound **53** significantly increased cellular iron levels, similar to the effect of Erastin, a well-recognized ferroptosis inducer (Figure 4A). By contrast, unlike Erastin, Compound **53** did not significantly increase cellular ROS levels, consistent with a previous report that NFS1 knockdown via siRNA did not affect ROS levels in lung cancer cells [16]. These results strongly indicate that Compound **53** is a selective inhibitor of human NFS1 and can suppress the proliferation of A549 cells by targeting NFS1 to disrupt Fe-S cluster biosynthesis.

Ferroptosis is an iron-dependent form of non-apoptotic cell death characterized by the toxic accumulation of lipid peroxides (LPO) [32]. In recent years, ferroptosis has been increasingly recognized for its potential as a therapeutic strategy in cancer treatment [33,34]. In this study, it was observed that treatment with Compound **53** increased cellular iron content but did not affect ROS levels. Interestingly, Compound **53**, in combination with 2-AAPA, an irreversible inhibitor of glutathione reductase (GR), elevated cellular ROS levels and induced ferroptotic cell death, as evidenced by an increase in lipid peroxidation (LPO) content (Figure 5D). These results highlight the potential of Compound **53** as a selective inhibitor for studying the biological roles of NFS1 and as a lead compound for the treatment of NFS1-related cancers. However, since Compound **53** showed only moderate inhibitory activity against human NFS1, further studies are required to explore its structurally optimized derivatives through chemical design and synthesis and to identify more potent compounds.

## 4. Materials and Methods

### 4.1. Compounds and Reagents

The anti-cysteine desulfurase NFS1 antibody and the anti-glyceraldehyde-3-phosphate dehydrogenase (GAPDH) antibody were purchased from ABclonal (Wuhan, China). All the compounds used for screening were obtained from Topscience Technology (Shanghai, China). 2-Acetylamino-3-[4-(2-acetylamino-2-carboxyethylsulfanylcarbonylamino) phenyl carbamoylsulfanyl] propionic acid (2-AAPA), DL-propargylglycine (PAG), aminooxyacetic acid (AOAA), and Erastin were purchased from Sigma-Aldrich (St. Louis, MO, USA). The fluorescent dye 2′,7′-dichlorodihydrofluorescein diacetate (DCFH-DA) used for reactive oxygen species (ROS) detection, the lipid peroxide (LPO) content assay kit, and the succinate dehydrogenase (SDH) activity assay kit were obtained from Solarbio (Beijing, China). The cell counting kit-8 (CCK-8) was purchased from Dojindo (Kumamoto, Japan). Fetal bovine serum was obtained from Biological Industries (Kibbutz Beit Haemek, Israel).

### 4.2. Protein Expression and Purification

Since human NFS1 requires the auxiliary protein ISD11 to stabilize its structure, it has been shown that catalytically active NFS1 can be prepared from *E. coli* cells by co-expressing NFS1 and ISD11 [35,36]. For the co-expression of NFS1 and ISD11, the NFS1 and ISD11 cDNA fragments were cloned from a human cDNA library. Primers were designed to generate a deletion of the first 55 amino acids of NFS1, allowing cloning into the *EcoR*I-*Not*I sites of the expression vector pACYCDuet-1. The resulting plasmid was designated pNFS1Δ1-55. Next, primers were designed to enable the cloning of ISD11 into the *Bgl*II-*Xho*I sites of the plasmid pNFS1Δ1-55. The resulting plasmid, designated pNFS1-ISD11, co-expresses NFS1 with an N-terminal His tag and ISD11 without a tag.

For the expression and purification of NFS1Δ1-55/ISD11 in *E. coli*, the plasmid pNFS1-ISD11 was transformed into *BL21*(DE3) cells. The cells were grown at 37 °C in 1 L cultures of LB medium containing chloramphenicol (50 μg/ml) until the optical density at 600 nm (OD_600_) reached 0.6–0.8. Protein expression was induced by adding 0.1 mM isopropyl β-D-1-thiogalactopyranoside (IPTG), followed by an additional 12 h of incubation at 25 °C. The cells were harvested by centrifugation at 8000 rpm for 10 min at 4 °C, then resuspended in lysis buffer containing 50 mM phosphate-buffered saline (PBS, pH 7.4), 500 mM NaCl, 100 mg/L lysozyme, 30 mM imidazole, and 1 mM phenylmethanesulfonyl fluoride (PMSF). The resuspended cells were incubated on ice for 1 h, followed by sonication using an Ultrasonic Cell Disruption System (Scientz, Ningbo, China). The supernatant was obtained by centrifugation at 12,000 rpm for 30 min at 4 °C and was loaded onto a HisTrap FF column (GE Healthcare, Chicago, MA, USA) pre-equilibrated with lysis buffer. The recombinant NFS1Δ1-55/ISD11 protein was eluted with 500 mM of imidazole in 50 mM of PBS (pH 7.4), and the collected fractions were desalted using a HiTrap Desalting column (GE Healthcare, Chicago, MA, USA) pre-equilibrated with 50 mM of phosphate buffer (pH 7.4). Fractions containing the recombinant NFS1Δ1-55/ISD11 protein were pooled and stored at −80 °C. The protein concentration was measured using a BCA protein assay reagent kit (TransGen Biotech, Beijing, China). Mutagenesis of NFS1 was performed using a TaKaRa MutanBEST Kit (Takara, Dalian, China). The expression and purification of the NFS1 mutants were carried out as described above for the wild-type protein. Additionally, the expression and purification of human cystathionine-β-synthase (CBS) and cystathionine-γ-lyase (CSE) were conducted as described previously [37].

### 4.3. Structure-Based In Silico Screening of Human NFS1 Inhibitors

Molecular docking-based virtual screening was performed using the Schrödinger release 2015-3 software package (Schrödinger, LLC, New York, NY, USA). The crystallographic structure of human NFS1 in complex with the cofactor PLP (PDB ID: 5WLW) was retrieved from the RCSB Protein Data Bank (PDB) (http://www.rcsb.org/, accessed on 29 October 2021). The Protein Preparation Wizard module in Schrödinger release 2015-3 was used to remove crystallographic water molecules, add missing side chains and hydrogen atoms, and optimize the protein structure [38]. Hydrogen bonds and protonation states were optimized using PROPKA at pH 7.0. A restrained minimization was performed with the OPLS3 force field to converge heavy atoms to a root-mean-square deviation (RMSD) of 0.3 Å. Residues within 5 Å of the substrate-binding active site in the crystal structure were defined as the binding site, where the docking grids were created. The ligand database was extracted from the commercial ChemDiv library (http://www.chemdiv.com/, accessed on 29 October 2021), which contains approximately 1.6 million molecules. The ChemDiv library was pre-filtered using Lipinski’s rule of five. Subsequently, the filtered compounds were prepared using the LigPrep panel with the OPLS3 force field [39]. Epik was employed to generate ionization states, tautomers, and stereoisomers at pH 7.0 ± 2.0 [40]. Three levels of molecular docking-based virtual screening (HTVS: High-Throughput Virtual Screening mode; SP: Standard Precision mode; and XP: Extra Precision mode) were sequentially conducted using the Glide module [41], resulting in the retrieval of the top-ranked 600 compounds based on Glide scores from the ChemDiv library. At each stage, only the top 10% scoring compounds were selected to advance to the next stage. When selecting candidate compounds, the following criteria were applied: (1) good water solubility, and (2) structural diversity. The top-ranked 283 molecules were clustered into 20 groups using the Functional-Class Fingerprints_6 (FCFP_6) algorithm. From these groups, 42 compounds with high docking scores were selected and purchased from TargetMol (Boston, MA, USA) for subsequent biological evaluation. As a result, two compounds (Compound **1** and Compound **37**) were identified as potential inhibitors of NFS1 activity.

To discover more potent NFS1 inhibitors, a similarity-based analog search was conducted to identify distinct chemotypes from these two inhibitors using pharmacophore models. Pharmacophore models for Compounds **1** and **37** were generated to establish a structure–activity relationship (SAR). The substructure search function embedded in the Molecular Operating Environment (MOE) 2022 software was employed to identify analogs with core scaffold architectures (Murcko frameworks) of the two promising NFS1 inhibitors within the ChemDiv library. A total of 383 compounds with pharmacophore fit scores of at least 2 were selected for further analysis. Finally, the top 41 compounds were chosen and purchased from TargetMol (Boston, MA, USA) to test their inhibitory activity against NFS1.

### 4.4. NFS1 Inhibition Assays

The activity of NFS1 was determined using a methylene blue assay as described previously, with some modifications [22]. Briefly, reaction mixtures (200 μL) containing 50 mM of Tris-HCl (pH 8.0), 10 μM of PLP, 1 mM of DTT, and 10 μg of NFS1 were preincubated at 37 °C for 3 min. The reaction was then initiated by adding 1 mM of cysteine. After 15 min of incubation at 37 °C, the reaction was quenched by adding 25 μL of 20 mM N,N-dimethyl-p-phenylenediamine in 7.2 M of HCl and 25 μL of 30 mM FeCl_3_ in 1.2 M of HCl. Subsequently, the reaction mixtures were incubated for another 15 min at 37 °C, followed by centrifugation at 12,000 rpm for 10 minutes to remove the precipitates. The absorbance of the supernatant was measured at 670 nm. A reaction mixture without the substrate cysteine was used as the negative control. The H_2_S concentration was calculated based on a standard curve prepared using various concentrations of sodium sulfide (Na_2_S).

For the NFS1 inhibition assays, all test compounds were dissolved in dimethyl sulfoxide (DMSO) to prepare 10 mM stock solutions, which were stored at −80 °C. The stock solutions were diluted to the appropriate concentrations with 50 mM of Tris-HCl (pH 8.0) as required for the experiments. A reaction mixture containing DMSO but no test compounds was used as a negative control. The dose-dependent effects of the inhibitors on NFS1 activity were determined using the methylene blue assay described above. The IC_50_ values of the inhibitors were calculated by adding different concentrations of the inhibitors to the enzyme reaction mixtures.

### 4.5. Selectivity Assay

The H_2_S-producing activity of CBS was measured as previously described [42]. The reaction mixture (200 μL) contained 50 mM of Hepes buffer (pH 7.4), 2 mM of homocysteine, 10 μM of PLP, 0.4 mM of lead nitrate, and 5 μg of CBS protein. It was preincubated at 37 °C for 3 min, and the reaction was initiated by the addition of 2 mM of cysteine. The reaction was monitored in a multifunctional microplate reader at 37 °C for 10 min. An extinction coefficient of 5500 M^−1^ cm^−1^ at 390 nm was used to calculate the lead sulfide concentration. The method used to determine CSE activity in the H_2_S-producing assay was identical to the CBS assay, except that 1 mM of cysteine was used as the sole substrate. The inhibitory activities of Compound **53** and Compound **56** on CBS and CSE were determined by adding different concentrations of the selected compounds to the respective enzyme reaction mixtures. Aminooxyacetic acid (AOAA), a widely used CBS inhibitor, and DL-propargylglycine (PAG), a selective CSE inhibitor, were used as positive controls, respectively.

### 4.6. Cell Culture and Cell Viability Assay

Human bronchial epithelial cells (BEAS-2B) and human lung adenocarcinoma A549 cells were purchased from the Institute of Biochemistry and Cell Biology, Chinese Academy of Sciences (Shanghai, China). BEAS-2B and A549 cells were cultured in DMEM supplemented with 10% (*v*/*v*) fetal bovine serum and 1% (*v*/*v*) penicillin–streptomycin solution at 37 °C in a humidified atmosphere containing 5% CO_2_. The half-maximal inhibitory concentration (IC_50_) values of the selected compounds against BEAS-2B and A549 cells were determined using the CCK-8 assay. Briefly, the cells were seeded into 96-well culture plates at a density of 5 × 10^3^ cells per well. After 12 h of culture, fresh medium containing varying concentrations of the selected compounds dissolved in DMSO was added for an additional 72-hour incubation. Subsequently, 10 μL of CCK-8 solution was added to each well, followed by a further 1.5-h incubation at 37 °C. The optical density at 450 nm (OD_450_) for each well was measured using a microplate reader. Medium containing DMSO alone was used as the control group. Cell viability was calculated as the percentage of the OD_450_ of the experimental group compared to that of the control group, which was set at 100%.

### 4.7. Knockdown of NFS1 by Lentivirus-Mediated RNA Interference

Small interfering RNAs (siRNAs) targeting human NFS1 were synthesized by Tsingke Biotech (Beijing, China) and transfected into A549 cells using HighGene plus Transfection reagent (ABclonal, Wuhan, China). The following siRNA sequences, 5′-GGUAUAUUUCCAUACUGAUTT-3′ and 5′-AUCAGUAUGGAAAUAUACCTT-3′, specific to human NFS1, were used for lentivirus packaging. Lentiviruses carrying NFS1 short hairpin RNA (shRNA) were synthesized by Hanbio Biotechnology (Shanghai, China). A549 cells, at 40–60% confluency, were infected with 15 μL of lentivirus and 8 μg/mL of polybrene for 48 hours, followed by selection with 10 μg/mL of puromycin (Solarbio, Beijing, China) for 7 days in a 6-well plate.

### 4.8. Immunoblotting

Cells were washed three times with pre-cooled PBS and then lysed in RIPA buffer (50 mM of Tris-HCl, pH 7.4, 150 mM of NaCl, 1% NP-40, 0.5% sodium deoxycholate) supplemented with 1 mM of PMSF. The supernatant was collected by centrifugation at 12,000 rpm for 10 min at 4 °C, and the protein concentration was measured using a BCA protein assay reagent kit (TransGen Biotech, Beijing, China). Proteins (50 μg per lane) were resolved by SDS-PAGE and then transferred to a polyvinylidene difluoride (PVDF) membrane. The membranes were incubated overnight at 4 °C with an anti-NFS1 antibody (ABclonal, Wuhan, China) diluted at 1:1000, followed by incubation with horseradish peroxidase (HRP)-conjugated secondary antibodies for 1 hour at room temperature. Subsequently, signals were visualized using an enhanced chemiluminescence (ECL) detection system (Bio-Rad, Hercules, CA, USA). Band intensity quantification was performed using ImageJ 1.53e software (National Institutes of Health, Bethesda, MD, USA). Glyceraldehyde-3-phosphate dehydrogenase (GAPDH) was used as a loading control.

### 4.9. Assays of Succinate Dehydrogenase and Aconitase Activity

The activity of succinate dehydrogenase (SDH) in cells was determined using an SDH activity assay kit (Solarbio, Beijing, China) according to the manufacturer’s instructions. The optical density at 600 nm (OD_600_) was measured using a multifunctional microplate reader in 96-well plates. One unit of SDH activity was defined as the amount of enzyme catalyzing the consumption of 1 nmol of 2,6-dichlorophenol indophenol per minute at 37 °C [43]. The activity of aconitase (ACO) was measured as previously described, with some modifications [44,45]. The cells were washed three times with pre-cooled PBS and lysed with lysis buffer containing 50 mM of Tris-HCl (pH 8.0), 0.2% Triton X-100, 0.6 mM of MnCl_2_, 5 mM of citrate, and 1 mM of PMSF. The aconitase activity was measured by monitoring the formation of cis-aconitate from isocitrate at 240 nm in 50 mM of Tris-HCl (pH 8.0) containing 20 mM of DL-trisodium isocitrate at 37 °C. An extinction coefficient of 3600 M^−1^ cm^−1^ at 240 nm was used to calculate the cis-aconitate concentration. One unit of ACO activity was defined as the amount of enzyme catalyzing the generation of 1 nmol of cis-aconitate per minute at 37 °C.

### 4.10. Measurement of Iron Elements

A549 cells were seeded in a 6-well plate and grown to 40–60% confluence. Compound **53** (50 μM) or Erastin (2.5 μM) was added to the culture medium, respectively, followed by incubation for 48 h. The cells were then washed three times with PBS and digested with 0.25% trypsin (Servicebio, Wuhan, China) for 2 min. The cells were divided into two equal groups: group A and group B. In group A, the cells were lysed with RIPA lysis buffer supplemented with 1 mM of PMSF to extract the total proteins. Protein concentrations were measured using a BCA protein assay reagent kit. In group B, the cells were treated with concentrated nitric acid at 70 °C for 2 h. Once the cells were completely nitrated, the temperature was increased to 150 °C until the nitric acid had completely evaporated. Subsequently, the samples were diluted to 10 mL with a solution containing 1% HNO_3_ and 0.1% KCl, and the iron levels were measured using atomic absorption spectroscopy (AAS; Analytik Jena AG, Jena, Germany) [46,47]. The iron content was calculated based on the standard curve, and the values were normalized to the protein concentration.

### 4.11. Images of Endogenous ROS in Live Cells

Endogenous ROS levels in A549 cells were detected using the fluorescent dye DCFH-DA [48,49]. A549 cells were cultured in DMEM supplemented with 10% fetal bovine serum at 37 °C in a humidified atmosphere containing 5% CO_2_. When the cells reached 40–60% confluence in 6-well plates, Compound **53** (50 μM), Erastin (2.5 μM), or 2-AAPA (20 μM) was added to the culture medium, followed by incubation for 48 h. Subsequently, the cells were washed three times with prewarmed PBS, and fresh medium containing 10 μM of DCFH-DA was added. After 30 min of incubation, the cells were washed again with prewarmed PBS, fresh medium was added, and the cells were imaged using an inverted fluorescence microscope (Leica, Wetzlar, Germany). Images of live cells were acquired in six different fields. ImageJ software was used to analyze the images.

### 4.12. Detection of Lipid Peroxide (LPO) Content

A549 cells were seeded in 10 cm dishes at a density of 2 × 10^6^ cells per dish and cultured until they reached 40–60% confluence. The cells were then treated with 2.5 μM of Erastin, 20 μM of 2-AAPA, or 50 μM of Compound 53 for 48 h. Lipid peroxidation (LPO) levels were measured using a Lipid Peroxide (LPO) Content Assay Kit (Solarbio, China) following the manufacturer’s instructions.

### 4.13. Molecular Docking and Molecular Dynamics Simulation

Molecular docking was performed using AutoDock Vina 1.1.2 [50] and the optimal docking pose was selected as the initial conformation for subsequent simulations. All molecular dynamics (MD) simulations were conducted using Gromacs 2019.6. For the NFS1 protein docked with Compound **53**, the Amber99SB-ILDN force field was applied to the protein [51], while the GAFF force field was used for Compound **53** [52]. ACPYPE (https://pypi.org/project/acpype/, accessed on 29 November 2024) was employed to generate the corresponding force field and topology files for Compound **53**. The simulation system was prepared in a cubic box filled with the TIP3P water model [53], with the box boundary set 10 Å away from NFS1. The system charges were neutralized using Na^+^ or Cl^−^ ions, followed by energy minimization using the steepest descent method. During the MD simulations, all relevant bonds were constrained using the LINCS algorithm, and electrostatic interactions were calculated with the Particle-Mesh Ewald (PME) method [54]. Temperature control was maintained at 310 K using the Berendsen thermostat [54], while pressure control was achieved using the Parrinello–Rahman barostat at 1 bar [55].

The system was equilibrated at the desired temperature and pressure upon the completion of the two equilibration phases. Subsequently, a 200 ns unrestrained MD simulation was performed. The trajectory files were generated using the GROMACS trjconv command after the MD simulations. The root-mean-square deviation (RMSD) values for the NFS1–Compound **53** complex were calculated using the GROMACS rms command. The binding free energy (Δ*G*_bind_) of Compound **53** with NFS1 was analyzed using the Molecular Mechanics–Poisson Boltzmann Surface Area (MM-PBSA) method [56]. The interactions between Compound **53** and NFS1 were visualized using PyMOL 3.8.5 [57,58].

### 4.14. Effects of Compound 53 on the Activity of Wild-Type and Mutant NFS1 Proteins

The dose-dependent effects of Compound **53** on the activity of wild-type and mutant NFS1 enzymes were determined using the H_2_S-producing assay described above. The IC_50_ values of Compound **53** against wild-type and mutant NFS1 enzymes were measured in the presence of 1 mM of cysteine, 1 mM of DTT, 10 μg of NFS1 protein, and 10 μM of PLP at a total volume of 200 μL. The reaction mixture containing DMSO without Compound **53** was used as a negative control.

### 4.15. Statistical Analysis

Data are presented as the means ± SD. Unpaired Student’s *t*-test and one-way analysis of variance (ANOVA) were performed to determine statistical significance using GraphPad Prism 8.0. A *p* value of <0.05 was considered statistically significant.

## 5. Conclusions

In summary, through molecular docking-based virtual screening and biological validation, Compound **53** was identified as a novel, selective inhibitor of human NFS1. The key residues involved in the binding of the inhibitor to NFS1 were identified through a combination of molecular docking and site-directed mutagenesis. Additionally, treatment with Compound **53** inhibited the proliferation of lung cancer cells and increased cellular iron levels by disrupting Fe-S cluster biogenesis. Compound **53** can be utilized to investigate the biological roles of NFS1 and can serve as a lead compound for developing treatments for NFS1-related cancers.

## Data Availability

Data are contained within the article.

## Supplementary Materials

The following supporting information can be downloaded at: https://www.mdpi.com/article/10.3390/ijms26062782/s1.

## Abbreviations

NFS1, cysteine desulfurase; Fe-S cluster, iron–sulfur cluster; CBS, cystathionine β-synthase; CSE, cystathionine γ-lyase; AOAA, aminooxoacetic acid; PAG, DL-propargylglycine; ACO, aconitase; SDH, succinate dehydrogenase; DMSO, dimethyl sulfoxide; ROS, reactive oxygen species; PLP, pyridoxal phosphate; LPO, lipid peroxidation; PMSF, phenylmethylsulfonyl fluoride; 2-AAPA, R,R′-2-Acetylamino-3-[4-(2-acetylamino-2-carboxyethylsulfanylcarbonylamino)phenyl carbamoylsulfanyl]propionic acid.
