# Supplementary material for: Identification of a Selective Inhibitor of Human NFS1, a Cysteine Desulfurase Involved in Fe-S Cluster Assembly, via Structure-Based Virtual Screening
Source: Int J Mol Sci. 2025 Mar 19;26(6):2782. doi: 10.3390/ijms26062782 (PMC11942905; doi:10.3390/ijms26062782)
Supplement: Supplementary file 1 [file ijms-26-02782-s001.zip › ijms-3519694-supplementary.pdf]

Supplementary Materials

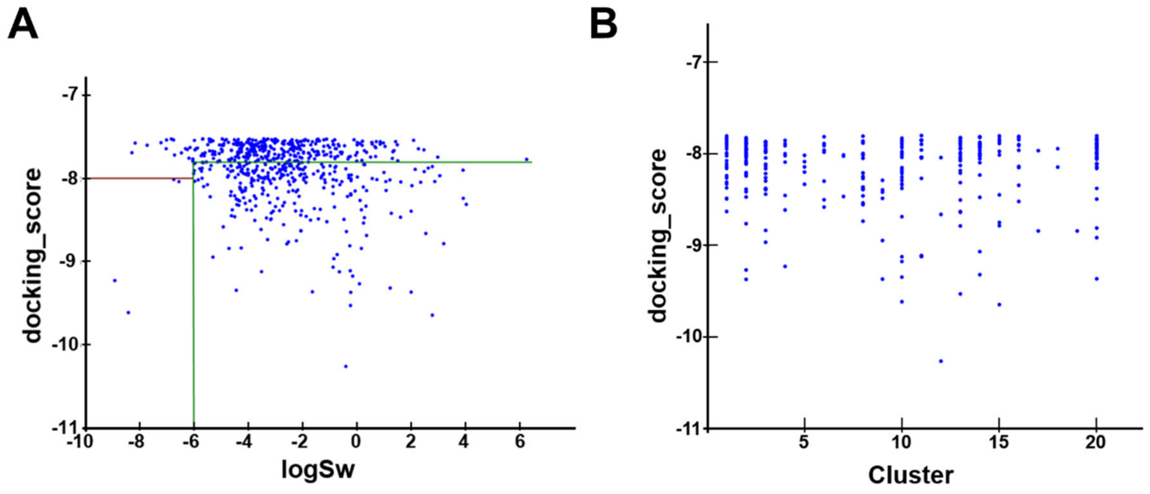

Figure S1. Analysis of virtual screening results. (**A**) A total of 283 small molecule compounds were screened based on solubility and affinity-based docking score data. The selected molecules exhibit a docking score < −7.8 and are water-soluble, with a logSw > −6. (**B**) The 283 molecules in the ChemDiv library are clustered into 20 groups based on the Functional-Class Fingerprints_6 (FCFP_6) algorithm.

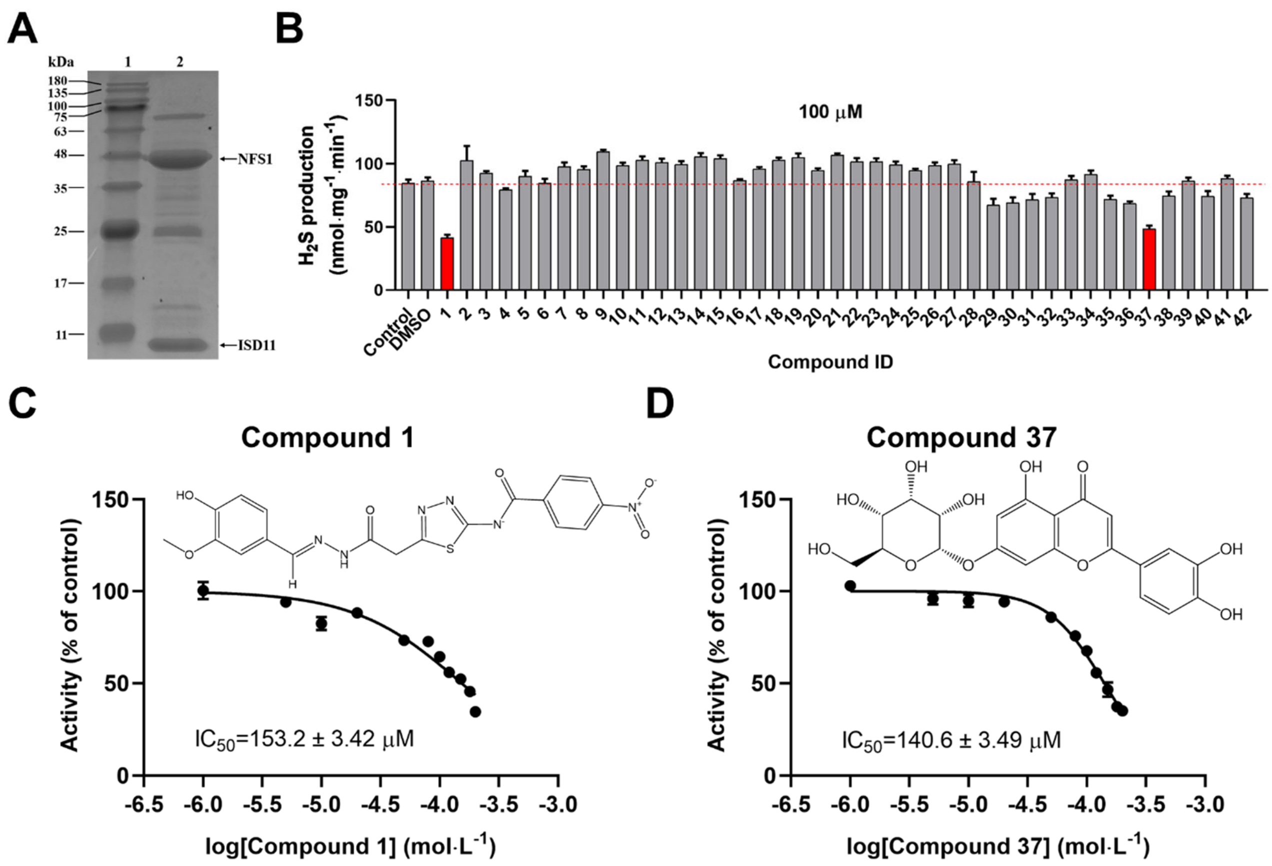

Figure S2. Preliminary evaluation of the effects of compounds obtained from virtual screening on NFS1 activity. (**A**) Purified human NFS1 was analyzed by SDS-PAGE. Lanes: 1. Marker; 2. Recombinant wild-type human NFS1. (**B**) Evaluation of the effects of compounds 1-42 obtained from virtual screening on NFS1 activity at a final concentration of 100 μM (n = 4). (**C**,**D**) Effects of Compounds **1** and **37** at various concentrations on NFS1 activity. The chemical structures of Compounds **1** and **37** are shown in the inset of Figure S2C and D, respectively. Data are presented as means ± SD (n = 4).

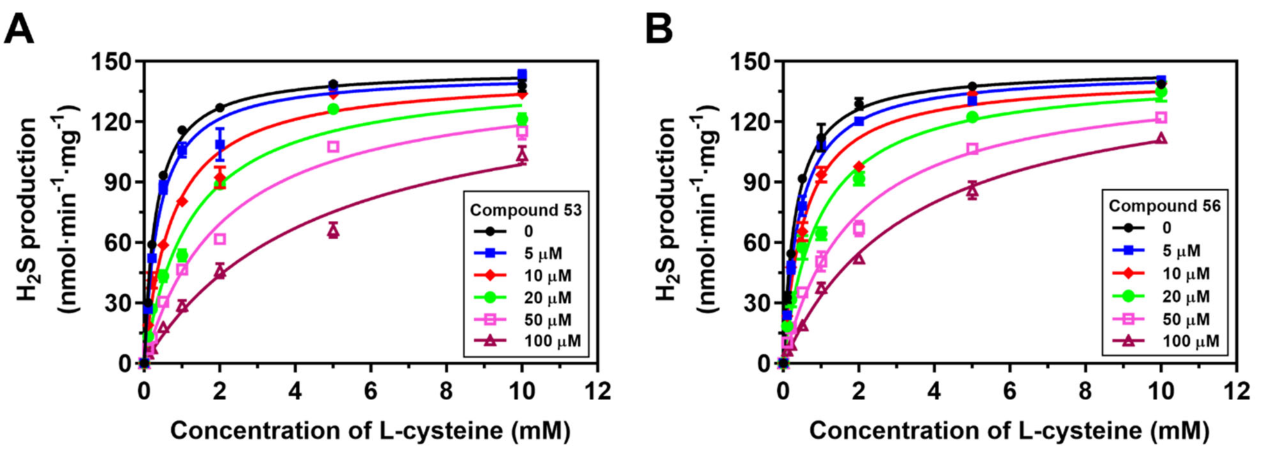

Figure S3. Evaluation of the effects of Compounds **53** (**A**) and **56** (**B**) at varying concentrations on NFS1 activity in the presence of different concentrations of the substrate L-cysteine.

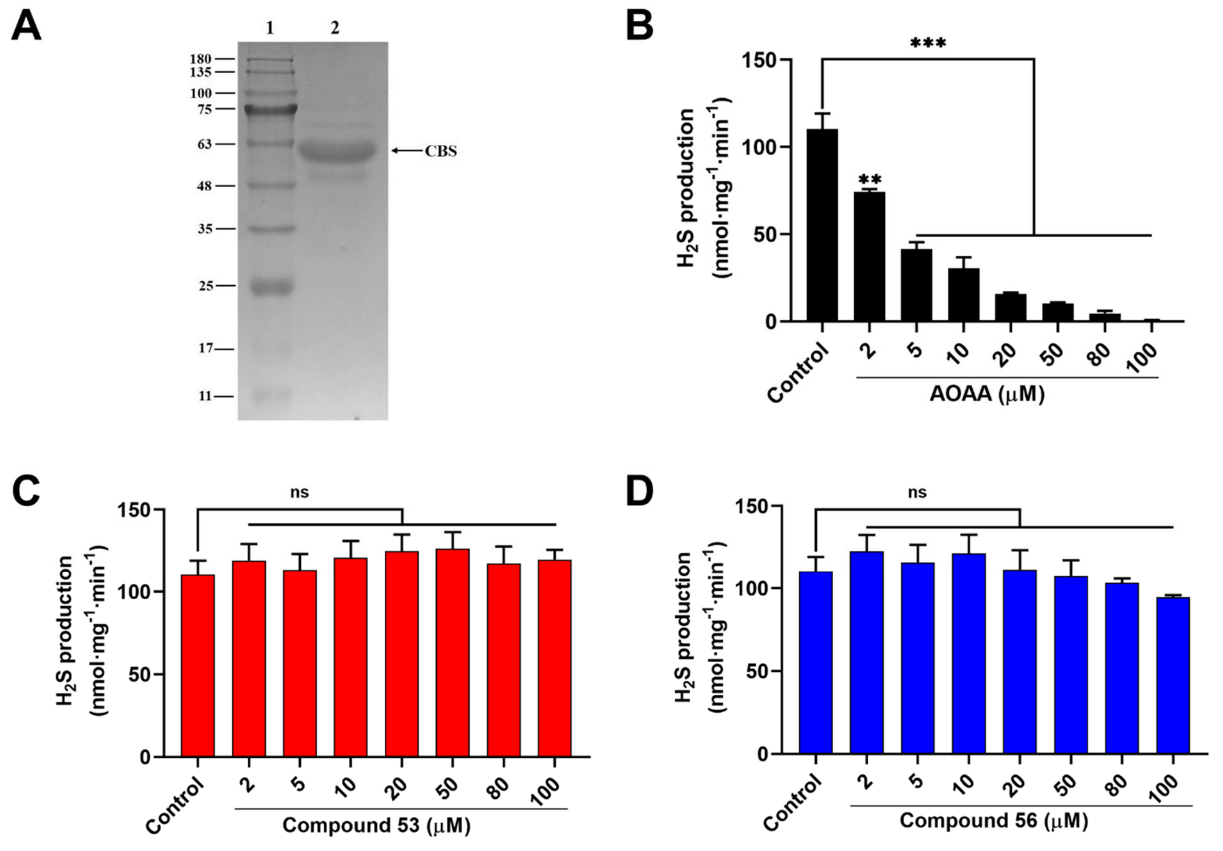

Figure S4. Evaluation of the selectivity of Compounds 53 and 56 towards NFS1 and another H_2S-producing protein, CBS. (**A**) Purified human CBS was analyzed by SDS-PAGE. Lanes: 1. Marker; 2. Recombinant wild-type human CBS. (**B**) Effect of AOAA at various concentrations on the H_2S-producing activity of CBS. (**C**,**D**) Effects of Compounds **53** and **56** at various concentrations on the H_2S-producing activity of CBS. Data are presented as means ± SD (n = 4). ** $p < 0.01$. *** $p < 0.001$.

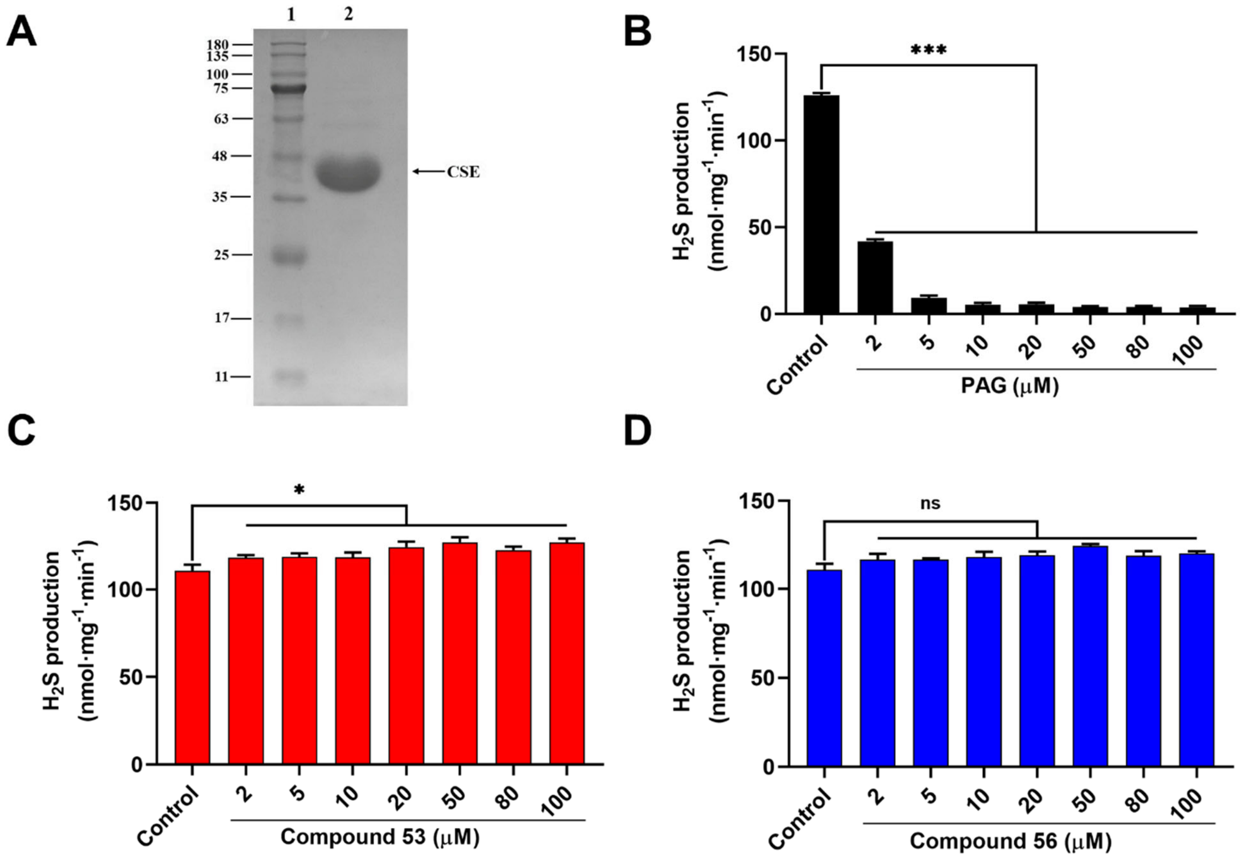

Figure S5. Evaluation of the selectivity of Compounds 53 and 56 towards NFS1 and another H_2S-producing protein, CSE. (**A**) Purified human CSE was analyzed by SDS-PAGE. Lanes: 1. Marker; 2. Wild-type human CSE. (**B**) Effect of PAG at various concentrations on the H_2S-producing activity of CSE. (**C**,**D**) Effects of Compounds **53** and **56** at various concentrations on the H_2S-producing activity of CSE. Data are presented as means ± SD (n = 4). * $p < 0.05$. *** $p < 0.001$.

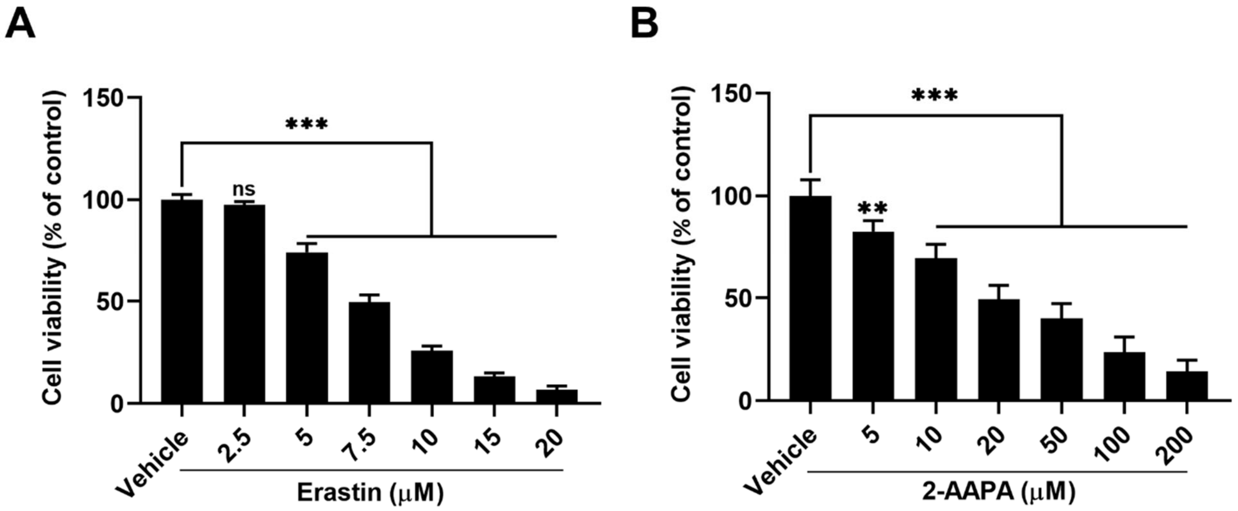

Figure S6. The effects of Erastin and 2-AAPA on the proliferation of A549 cells. (**A**) Cell viability assays of A549 cells treated with varying concentrations of Erastin. (**B**) Cell viability assays of A549 cells treated with varying concentrations of 2-AAPA. Data are presented as means ± SD (n = 6). ** $p < 0.01$. *** $p < 0.001$.

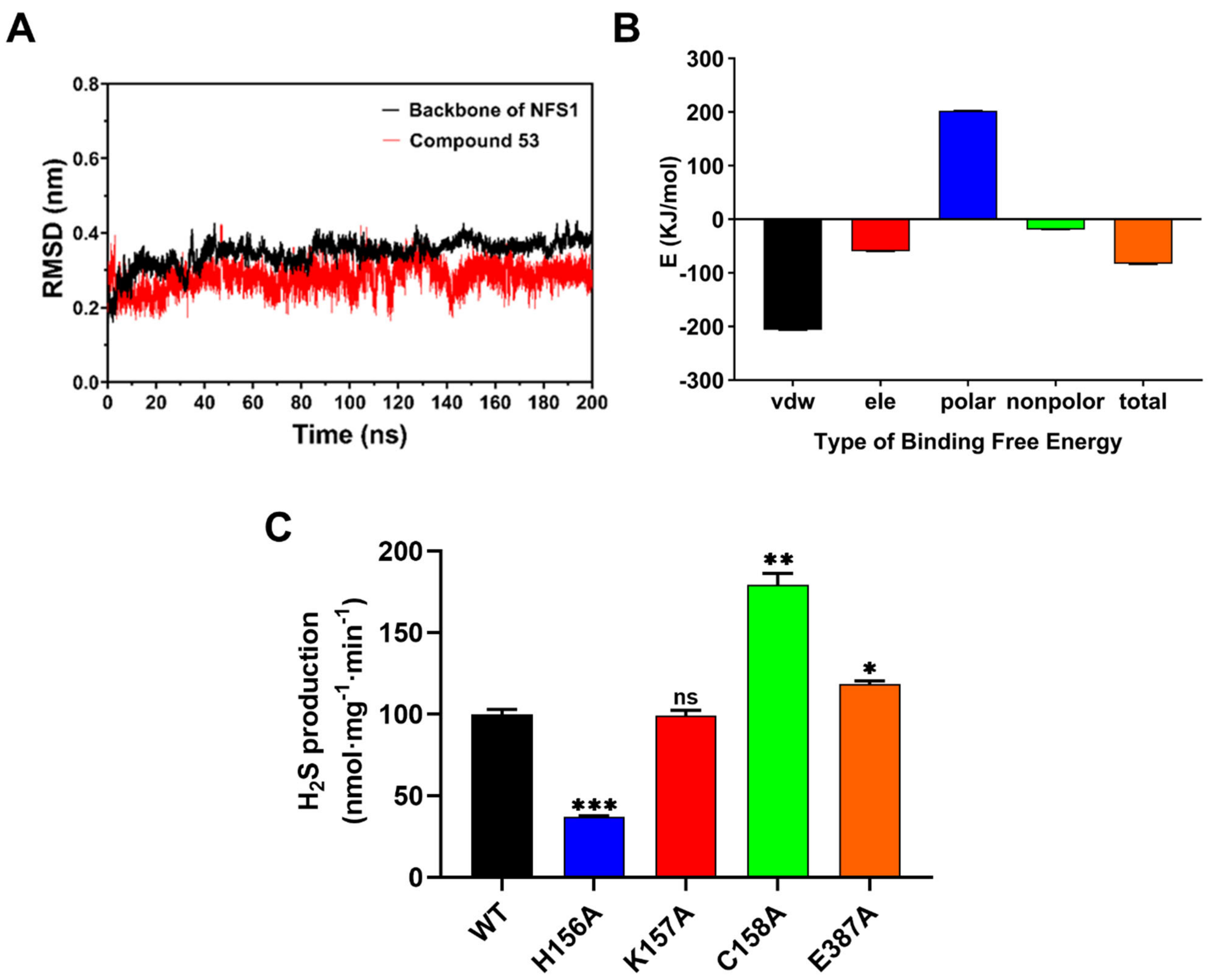

Figure S7. The molecular dynamics (MD) simulation of the NFS1-Compound 53 system. (**A**) RMSD change profiles of the complex and free protein systems over 200 ns of dynamic simulation. (**B**) The binding free energy between Compound **53** and NFS1. (**C**) The activities of the wild-type and four mutant NFS1 proteins were determined using the H_2S-producing assay (n = 4). Data are presented as means ± SD. * $p < 0.05$. ** $p < 0.01$. *** $p < 0.001$.

Table S1. Michaelis-Menten kinetic parameters of Compound **53** and Compound **56** against NFS1.

Inhibitor	Concentration (μM)	K_m (mM)	V_{max} (nmol·min^{-1}·mg^{-1})	Inhibition Type	K_i (μM)
Compound **53**	0	0.3 ± 0.01	145.9 ± 1.42	Competitive	6.4 ± 0.48
	5	0.4 ± 0.03	144.2 ± 2.81		
	10	0.7 ± 0.07	143.3 ± 4.06		
	20	1.3 ± 0.13	144.1 ± 4.88		
	50	2.1 ± 0.18	143.5 ± 4.31		
	100	4.6 ± 0.61	143.8 ± 8.75		
Compound **56**	0	0.3 ± 0.01	146.2 ± 1.36	Competitive	9.6 ± 0.57
	5	0.4 ± 0.02	145.3 ± 1.66		
	10	0.5 ± 0.05	142.3 ± 3.59		
	20	0.9 ± 0.09	143.9 ± 4.09		
	50	1.9 ± 0.14	143.7 ± 3.84		
	100	3.4 ± 0.21	148.3 ± 3.79		
